# APC Activation Restores Functional CD4^+^CD25^+^ Regulatory T Cells in NOD Mice that Can Prevent Diabetes Development

**DOI:** 10.1371/journal.pone.0003739

**Published:** 2008-11-17

**Authors:** Jean N. Manirarora, Michele M. Kosiewicz, Sarah A. Parnell, Pascale Alard

**Affiliations:** 1 Department of Microbiology and Immunology, University of Louisville, Health Sciences Center (HSC), Louisville, Kentucky, United States of America; 2 Department of Pathology, St. Jude Children's Research Hospital, Memphis, Tennessee, United States of America; New York University School of Medicine, United States of America

## Abstract

**Background:**

Defects in APC and regulatory cells are associated with diabetes development in NOD mice. We have shown previously that NOD APC are not effective at stimulating CD4^+^CD25^+^ regulatory cell function *in vitro*. We hypothesize that failure of NOD APC to properly activate CD4^+^CD25^+^ regulatory cells *in vivo* could compromise their ability to control pathogenic cells, and activation of NOD APC could restore this defect, thereby preventing disease.

**Methodology/Principal Findings:**

To test these hypotheses, we used the well-documented ability of complete Freund's adjuvant (CFA), an APC activator, to prevent disease in NOD mice. Phenotype and function of CD4^+^CD25^+^ regulatory cells from untreated and CFA-treated NOD mice were determined by FACS, and *in vitro* and *in vivo* assays. APC from these mice were also evaluated for their ability to activate regulatory cells *in vitro*. We have found that sick NOD CD4^+^CD25^+^ cells expressed Foxp3 at the same percentages, but decreased levels per cell, compared to young NOD or non-NOD controls. Treatment with CFA increased Foxp3 expression in NOD cells, and also increased the percentages of CD4^+^CD25^+^Foxp3^+^ cells infiltrating the pancreas compared to untreated NOD mice. Moreover, CD4^+^CD25^+^ cells from pancreatic LN of CFA-treated, but not untreated, NOD mice transferred protection from diabetes. Finally, APC isolated from CFA-treated mice increased Foxp3 and granzyme B expression as well as regulatory function by NOD CD4^+^CD25^+^ cells *in vitro* compared to APC from untreated NOD mice.

**Conclusions/Significance:**

These data suggest that regulatory T cell function and ability to control pathogenic cells can be enhanced in NOD mice by activating NOD APC.

## Introduction

Dysregulation of the immune response in NOD mice plays a major role in the induction of type 1 diabetes [1], [2]. NOD mice are defective in both central [3] and peripheral tolerance [1]. In addition, antigen-presenting cells (APC) in NOD mice exhibit various defects, including differentiation, antigen presentation and co-stimulatory molecule expression [4]–[7].

It is now well established that CD4^+^CD25^+^ regulatory T cells, which account for 5–10% of CD4^+^ cells in healthy mice and humans, control the development of many autoimmune diseases. Their mechanisms of action are still controversial [8], however, these cells absolutely require activation [9], [10], presumably by APC, to function *in vitro* and probably *in vivo*. The transcription factor, Foxp3 appears to be critical for CD4^+^CD25^+^ regulatory T cell development and function. Foxp3-deficient mice do not develop functional CD4^+^CD25^+^ regulatory T cells, and ectopic expression of Foxp3 in naive T cells converts them into cells with regulatory function [11]–[13]. Studies using transgenic mice expressing GFP under control of the Foxp3 promoter has confirmed the relationship between Foxp3 and regulatory cell function [8], [14]. Interestingly, a recent study has found a correlation between levels of Foxp3 expression and regulatory function [15], suggesting that failure to maintain optimal Foxp3 expression could compromise regulatory cell function and lead to autoimmune disease development.

Although controversial, the percentage of CD4^+^CD25^+^ regulatory T cells has been shown in some studies to be reduced in NOD mice [16]–[18] and patients with type 1 diabetes [19]. On the other hand, there is very clear evidence that this regulatory population is functionally defective in mice and patients with type 1 diabetes [20]–[22]. We have shown previously that it is not the NOD CD4^+^CD25^+^ regulatory cells themselves that are defective in assays for regulatory function, but the NOD APC that are defective in their ability to stimulate CD4^+^CD25^+^ regulatory function [16]. We speculated that this may also be the case *in vivo*, and that stimulation of NOD APC *in vivo* could enhance their ability to activate regulatory T cells and thusly, prevent disease. The ability of complete Freund's adjuvant (CFA), a potent activator of APC, to prevent type 1 diabetes in NOD mice is well-established, and although protection has been attributed to a variety of different cells, including suppressor T cells, NK cells and suppressor macrophages [23]–[25], the precise mechanism is unknown. In the present study, we investigated whether CFA could act at least in part by enhancing the ability of NOD APC to activate NOD CD4^+^CD25^+^ regulatory cells that can control autoreactive T cells and prevent disease in NOD mice.

## Methods

### Mice

C57BL/6 and NOD female mice from 6 to 34 weeks of age (Jackson Laboratory, Bar Harbor, ME), and 8-week-old female OVE26 and FVB mice (gift from Dr. Paul Epstein, University of Louisville, KY) were maintained under specific pathogen-free conditions as described in the Institutional Animal Care and Use Committee guidelines.

### Antibodies and flow cytometry

APC-anti-CD25, PerCP-anti-CD4, PE-anti-CD103 antibodies were purchased (BD Pharmingen, San Diego, CA). One million cells were incubated with Fc block and labeled with antibodies for 20 min in DPBS 1% FCS, 0.1% NaN0_3_, and washed twice. For membrane-bound TGFβ, cells were labeled with biotinylated anti-TGFβ antibody (R&D, Minneapolis, MN) in buffer containing BSA followed by streptavidin-PE (BD, San Diego, CA). For Foxp3 and granzyme B, cells pooled from multiple culture wells were intracellularly labeled with either PE-anti-Foxp3 or anti-granzyme B antibodies (eBioscience, San Diego, CA) according to the manufacturer's instructions. Cells were analyzed by FACS® using a FACScalibur (Becton Dickinson, Palo Alto, CA).

### Cell isolation and purification

Pancreatic cells were obtained by successive digestion at 37°C in 1 mg/ml, 0.5 mg/ml, and 0.25 mg/ml collagenase D for 15 min, 10 min and 6 min, respectively. CD4 cells from lymphoid organs were enriched using CD4 cell-enrichment columns (R&D, Minneapolis, MN), then CD25^+^ (purity consistently >90%) and CD25^−^ cells purified using PE-anti-CD25 antibody and anti-PE magnetic beads (Miltenyi Biotech, Auburn, CA) as per the manufacturer's instructions. For some experiments, CD4^+^CD25^+^ regulatory cells were sorted (MoFlo®, DakoCytomation, Fort Collins, CO) to >95% purity.

### T cell-depletion of spleen cells

**S**pleen cells were incubated in lysis buffer (RPMI 1640 with 25 mM Hepes and 0.3% BSA) containing anti-mouse CD90 antibody (Cedarlane, Hornby, Ontario, Canada), then with Low-Tox-M rabbit complement (1∶10; Cedarlane, Hornby, Ontario, Canada). The purity of the CD3^−^ cells was consistently >95%.

### Cell culture

For the T cell suppression assay, CD4^+^CD25^−^ T responder cells (2.5×10^4^/well in 96-well round-bottom plates) were cultured in complete media (RPMI 1640, 10% heat-inactivated FCS, 2 mM glutamine, 10 mM HEPES, 100 U/ml penicillin G sodium, 100 µg/ml streptomycin sulfate, and 1×10^−5^ M 2-mercaptoethanol) with irradiated spleen cells (APC; 2×10^5^/well), anti-CD3 antibody (0.5 µg/ml), and with or without CD4^+^CD25^+^ regulatory T cells (2.5×10^4^/well) for 4 days at 37°C in 5% CO_2_. Cells were pulsed with [^3^H] thymidine (0.5 µCi) for the last 18 hrs. Percent inhibition was calculated as [(1−(responder−regulatory cell cpm/responder cell cpm))×100]. To evaluate Foxp3 maintenance or granzyme B induction *in vitro*, CD4^+^CD25^+^ regulatory T cells from multiple mice were pooled and sorted, and cultured in multiple wells (2–4×10^4^ cells/well) in complete media for 18 or 40 hrs with irradiated T cell-depleted spleen cells (APC; 1×10^5^) and anti-CD3 antibody (0.5 µg/ml).

### Treatment of mice with CFA

Complete Freund's adjuvant (CFA) was prepared using non-viable desiccated *Mycobacterium tuberculosis* (H37 RA, Difco Laboratories, Detroit, MI) at 3, 1 or 0.5 mg/ml in PBS and emulsified in incomplete Freund's Adjuvant (IFA; Sigma-Aldrich, St Louis, MO). NOD mice were injected subcutaneously at 6–8 weeks of age with 100 µl of CFA.

### Adoptive transfer assay and assessment of diabetes

Pancreatic LN CD4^+^ cells were sorted and transferred (0.4×10^6^) by i.v. injection into 4 wk-old female NOD mice. In some experiments, CD4^+^ cells were first depleted of CD25^+^ cells by incubation with anti-CD25 antibody (7D4) and low-Tox rabbit complement (Cedarlane, Hornby, Ontario, Canada). Blood glucose was monitored weekly until 30 weeks of age, and mice were considered diabetic when glucose levels were >300 mg/dl for two consecutive weeks.

### mRNA extraction and real-time PCR

Total mRNA was extracted from CD4^+^CD25^+^ and CD4^+^CD25^−^ T cells sorted from lymphoid organs (purity >98%) and reverse transcribed using Picopure RNA isolation (Arcturus, Mountain View, CA) and a Taq Man reverse transcriptase kit (Applied Biosystems, Foster City, CA). cDNA was amplified in duplicate by real-time PCR using a SYBR Green PCR kit (Applied Biosystems) and glyceraldehyde-3-phosphate dehydrogenase (GAPDH) (5′-GGAGCGAGACCCCACTAACA-3′ and 5′-ACATACTCAGCACCGGCCTC-3′) and Foxp3 (5′-CCCACCTACAGGCCCTTCTC-3′ and 5′-GGCATGGGCATCCACAGT-3′) primers. Foxp3 mRNA amounts were normalized to GAPDH mRNA amounts, and fold increase compared to CD4^+^CD25^−^ T cells is represented.

### Statistical analysis

Data were analyzed using either the student's t test, or ANOVA and the Turkey-Kramer multiple comparisons test, Fisher's exact test or Mann-Whitney test. Each experiment was repeated with reproducible results 2–4 times. One representative experiment is shown in each figure.

## Results

### CD4^+^CD25^+^ T cells from NOD mice express lower levels of Foxp3

**Figure 1:**
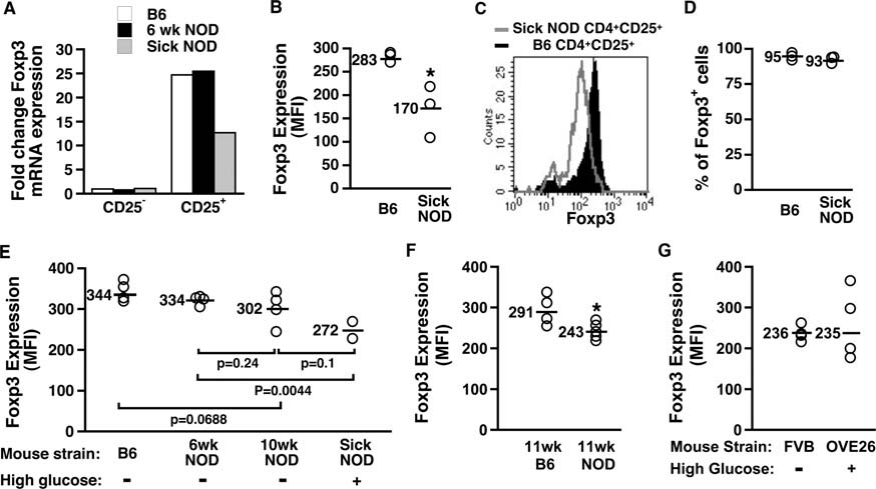
Foxp3 expression is decreased in CD4^+^CD25^+^ cells from NOD mice. Cells from lymphoid organs were harvested from 6-week-old NOD, or 15–20 week-old B6 and Sick NOD mice and pooled. CD4^+^CD25^−^ and CD4^+^CD25^+^ cells were sorted (>95% purity), and mRNA was isolated and real-time PCR performed using Foxp3 and GAPDH primers (A). Cells from lymphoid organs were harvested from 15–20 week-old B6 and Sick NOD mice (B–D); or 6 and 10-week-old NOD and B6 mice or 15–20 week-old Sick NOD mice (E); or 11 week-old B6 and NOD mice (F); or 8 week-old FVB wild-type (control) and OVE26 transgenic mice (G). Samples from individual mice were labeled with anti-CD4, CD25 and Foxp3 antibodies, and CD4^+^CD25^+^ cells were gated and analyzed for Foxp3 expression (% and MFI) by FACS® (B–G). Each point represents an individual animal. Blood glucose levels were evaluated weekly beginning at 12 weeks of age, and mice were considered diabetic (Sick) when glucose levels reached 300 dl/ml and were maintained for at least two consecutive weeks. An * indicates a significant difference from B6 controls at *p*<0.05.

We have found previously that APC from NOD mice are unable to activate CD4^+^CD25^+^ regulatory cells properly *in vitro* [16]. We speculated that NOD APC may exhibit a similar defect *in vivo* in NOD mice, which may be reflected by lower Foxp3 expression in CD4^+^CD25^+^ cells. We first examined Foxp3 expression in freshly harvested CD4^+^CD25^+^ cells from non-sick 6 week-old NOD, Sick (>300 dl/ml blood glucose for two consecutive weeks) 15–20 week-old NOD and age-matched B6 (control) mice. CD4^+^CD25^+^ cells from lymphoid organs of B6, Non-Sick and Sick NOD mice were labeled with anti-CD4 and anti-CD25 antibodies and either sorted (>95% purity) and Foxp3 mRNA quantified by real-time PCR, or also labeled with anti-Foxp3 antibody and analyzed by FACS®. Interestingly, Foxp3 mRNA and protein levels were significantly decreased in CD4^+^CD25^+^ cells from Sick NOD compared to B6 mice (Fig. 1A–C), although the percentages of CD4^+^CD25^+^ cells expressing Foxp3 were identical (Fig. 1D). We next evaluated the age at which decreases in Foxp3 expression can first be detected in NOD mice. We found that the level of Foxp3 expression in CD4^+^CD25^+^ cells from 6 week-old normoglycemic NOD mice was similar to that of B6 mice, but statistically different from Sick NOD mice (Fig. 1E). In contrast, CD4^+^CD25^+^ cells from 10 week-old pre-diabetic NOD mice (i.e., before elevated glucose can be detected in either urine or blood) expressed Foxp3 at a level that was not statistically different from that of 15–20 week-old Sick NOD mice. However, Foxp3 levels in CD4^+^CD25^+^ cells from 10-week-old NOD mice were also not statistically different from that of either 6 week-old NOD or B6 mice (Fig. 1E), suggesting that by 10 weeks of age CD4^+^CD25^+^ cells may be just beginning to show signs of lower Foxp3 expression. By 11 weeks of age, Foxp3 expression was significantly decreased in pre-diabetic NOD mice compared to B6 mice (Fig. 1F). Taken together, these data indicate that the decrease in Foxp3 expression may begin several weeks before disease onset, i.e., in our facility at about 10–12 weeks of age, and suggest that high levels of glucose may not affect Foxp3 expression. To directly evaluate the impact that high glucose has on Foxp3 expression, we next analyzed CD4^+^CD25^+^ cells from transgenic mice that were rendered chronically hyperglycemic beginning shortly after birth by rat insulin promoter driven expression of calmodulin (OVE26) [26] and subsequent β cell damage. CD4^+^CD25^+^ cells from eight week-old normoglycemic wild-type (FBV strain) and hyperglycemic OVE26 transgenic mice were analyzed for Foxp3 expression, and were found to express comparable Foxp3 levels (Fig. 1G). We concluded from these results that Foxp3 expression by CD4^+^CD25^+^ regulatory T cells is lower in diabetes-prone (NOD) than diabetes-resistant (B6) mice, and the lower expression of Foxp3 by NOD CD4^+^CD25^+^ cells is unlikely to be due to exposure to high glucose levels, although we cannot rule out a possible role for other metabolites in NOD mice.

### NOD APC are less effective than B6 APC in maintaining Foxp3 expression in CD4^+^CD25^+^ T cells *in vitro*

To begin to evaluate the possibility that the lower Foxp3 expression in fresh NOD CD4^+^CD25^+^ T cells (Fig. 1) may be due to decreased ability of NOD APC to maintain Foxp3 expression *in vivo*, we developed an assay to evaluate the ability of NOD APC to maintain Foxp3 expression *in vitro*. CD4^+^CD25^+^ cells from B6 mice were cultured overnight in the presence of T cell-depleted irradiated splenic cells (APC) pooled from B6 mice, and anti-CD3 antibody. The following day, the wells containing CD4^+^CD25^+^ T cells were pooled and Foxp3 expression was evaluated. As shown in Table 1, CD4^+^CD25^+^ T cells stimulated with APC and anti-CD3 expressed higher levels of Foxp3 (MFI) than unstimulated cells, suggesting that in the absence of APC-mediated activation optimal Foxp3 expression by CD4^+^CD25^+^ T cells is not maintained. Further, these data indicate that this assay can be used to evaluate APC efficacy in maintaining Foxp3 expression. In the next experiment, irradiated T cell-depleted spleen cells (APC) were pooled from Sick NOD or age-matched B6 mice, and used in cultures with NOD or B6 CD4^+^CD25^+^ T cells and anti-CD3 antibody. After overnight culture, cells were collected and CD4^+^CD25^+^ T cells evaluated for Foxp3 expression. Although there were no differences in the percentages of CD4^+^CD25^+^ cells expressing Foxp3 (Table 2, third column), the level of Foxp3 (MFI; Table 2, fourth column) expressed by either B6 or NOD CD4^+^CD25^+^ cells stimulated with NOD APC (Table 2, third & fifth rows, respectively) was decreased compared to B6 or NOD CD4^+^CD25^+^ cells stimulated with B6 APC (Table 2, second & fourth rows, respectively). The magnitude of differences in Foxp3 expression between NOD CD4^+^CD25^+^ cells stimulated with NOD and B6 APC *in vitro* was very similar to the differences between freshly harvested CD4^+^CD25^+^ cells from NOD and B6 mice (Fig. 1C), suggesting that ineffective or sub-optimal stimulation by NOD APC may be responsible for the lower expression of Foxp3 in NOD CD4^+^CD25^+^ cells *in vivo*. We also compared the ability of 11 week-old pre-diabetic NOD APC and Sick NOD APC to maintain Foxp3 expression *in vitro*. Interestingly, the level of Foxp3 expressed by NOD CD4^+^CD25^+^ cells stimulated with APC from pre-diabetic NOD mice was lower compared to cells stimulated with APC from B6 mice, but higher than cells stimulated with APC from Sick NOD mice (Table 3). These data suggest that APC from pre-diabetic mice may also start to exhibit a decreased ability to maintain Foxp3 expression *in vitro.* Taken together, these data support the possibility that NOD APC are less effective at maintaining Foxp3 expression by CD4^+^CD25^+^ T cells in NOD mice.

**Table 1:** *In vitro* assay to evaluate APC-mediated maintenance of Foxp3 expression by CD4^+^CD25^+^ T cells.

APC	CD4^+^CD25^+^ cells	% Foxp3^+^	Foxp3 MFI
none	B6	95	199
B6	B6	92	284

CD4^+^CD25^+^ T cells from 12–15 week-old B6 mice were cultured overnight in multiple wells, either alone or with B6 irradiated T-cell depleted spleen cells (APC) and anti-CD3 antibody. Cells were pooled and labeled with anti-CD4, CD25 and anti-Foxp3 antibodies. CD4^+^CD25^+^ T cells and APC were pooled from 3 mice. Cells were analyzed for Foxp3 expression by FACS® after gating on CD4^+^CD25^+^ cells. Representative results from one of two experiments are shown.

**Table 2:** APC from NOD mice are less efficient at maintaining Foxp3 expression *in vitro*.

APC	CD4^+^CD25^+^ cells	% Foxp3^+^	Foxp3 MFI
B6	B6	90	314
NOD	B6	92	249
B6	NOD	92	302
NOD	NOD	88	214

CD4^+^CD25^+^ T cells from 12–15 week-old B6 or Sick NOD mice were cultured overnight in multiple wells, either alone or with B6 or Sick NOD irradiated T-cell depleted spleen cells (APC) and anti-CD3 antibody. Cells were pooled and labeled with anti-CD4, anti-CD25 and anti-Foxp3 antibodies. CD4^+^CD25^+^ T cells and APC were pooled from 3 mice. Cells were analyzed for Foxp3 expression by FACS® after gating on CD4^+^CD25^+^ cells. Representative results from one of four experiments are shown.

**Table 3:** APC from pre-diabetic and Sick NOD mice are less efficient at maintaining Foxp3 expression *in vitro*.

APC	CD4^+^CD25^+^ cells	% Foxp3^+^	Foxp3 MFI
B6	NOD	94	232
Pre-diabetic NOD	NOD	98	182
Sick NOD	NOD	94	147

CD4^+^CD25^+^ T cells from 12–15 week-old Sick NOD mice were cultured overnight in multiple wells, either alone or with irradiated T-cell depleted spleen cells (APC) from B6, pre-diabetic (11 week-old) NOD or Sick NOD mice and anti-CD3 antibody. Cells were pooled and labeled with anti-CD4, anti-CD25 and anti-Foxp3 antibodies. CD4^+^CD25^+^ T cells and APC were pooled from 3 mice. Cells were analyzed for Foxp3 expression by FACS® after gating on CD4^+^CD25^+^ cells. Representative results from one of two experiments are shown.

### Prevention of diabetes following *in vivo* treatment with CFA involves induction/activation of CD4^+^CD25^+^ regulatory cells

**Figure 2:**
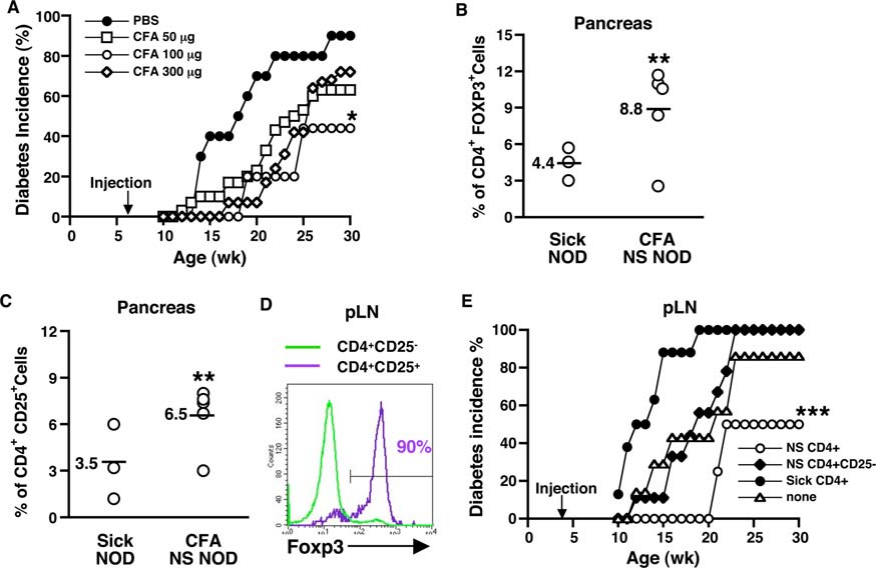
Disease-free CFA-treated NOD mice possess potent regulatory cells. Three different doses of CFA containing 50, 100 or 300 µg of *Mycobacterium tuberculosis* or PBS were administered s.c. to 6 week-old NOD mice. An * indicates a significant difference from the PBS control at *p*<0.03 (A). Pancreata were harvested from 34 week-old Sick (Sick) or CFA-treated Non-Sick (NS) NOD mice and digested with collagenase. Cells from individual mice were labeled with anti-CD4, CD25 antibodies, and Foxp3 antibodies and analyzed by FACS®. Each point represents an individual animal. An ** indicates a significant difference from the Sick NOD group at *p*<0.05 (B & C). Pancreatic LN cells were harvested from 30-week-old CFA-treated Non-Sick NOD mice, and labeled with anti-CD4, CD25 and Foxp3 antibodies, and analyzed by FACS® (D). Pancreatic LN cells from 30-week-old Sick or CFA-treated Non-Sick (NS) were pooled, CD4^+^ cells purified and 0.4×10^6^ cells transferred into 4-wk-old NOD mice. Or purified CD4^+^ cells from pancreatic LN from 30-week-old CFA-treated Non-Sick (NS) were depleted of CD25^+^ cells (NS CD4^+^CD25^−^) and 0.4×10^6^ of these CD4^+^CD25^−^ cells injected into 4-wk-old NOD mice. Blood glucose was monitored weekly and diabetes incidence determined. An *** indicates a significant difference from the untreated controls at *p*<0.005 (E).

Many studies have shown that s.c. injection of complete Freund's adjuvant (CFA) can delay onset and decrease incidence of diabetes in NOD mice [23]–[25]. CFA contains desiccated *Mycobacterium tuberculosis* (MT), a potent activator of APC. We hypothesized that CFA prevents diabetes in NOD mice by activating APC that subsequently activate regulatory cells. In initial experiments, different doses of CFA were evaluated for efficacy in preventing disease. As shown in Figure 2A, a single s.c. injection (100 µl) of CFA containing 100 µg of MT at 6 weeks of age, delayed diabetes onset by about 8 weeks or more, and 60% of the mice remained disease-free through at least 30 weeks of age. Injection of a lower (50 µg) or higher dose (300 µg) of MT was less protective, and therefore, the 100 µg dose of MT was used for all of the following experiments. These data may indicate that MT can influence the balance between pathogenic cell and regulatory cell activation, i.e., a higher dose of MT may tilt the balance in favor of the pre-existing pathogenic cells, whereas an intermediate dose may favor regulatory cells. In support of this hypothesis, we have found that the ratio of anti-inflammatory versus pro-inflammatory cytokines produced by APC is highly dependent on the dose and type of stimuli, and can in turn influence disease development, i.e., prevention versus exacerbation of diabetes, respectively ([27] and unpublished data).

We next examined the mechanism of CFA-mediated protection in NOD mice. Because 40% of CFA-treated NOD mice will eventually develop diabetes and all mice that are going to develop diabetes will do so by 30 weeks of age, we analyzed Sick and non-Sick NOD mice that were >30 weeks of age. Lymphocytic infiltrates in the pancreas of 34-week-old Non-Sick (NS) CFA-treated and Sick NOD mice were evaluated for Foxp3-expressing cells by FACS. The pancreas of Non-Sick CFA-treated mice contained significantly higher percentages of CD4^+^Foxp3^+^ and CD4^+^CD25^+^ cells than Sick NOD mice (Fig. 2B & C), suggesting that CD4^+^CD25^+^Foxp3^+^ regulatory cells could play a role in CFA-mediated prevention of disease. To investigate this possibility, pancreatic LN from Non-Sick (NS) CFA-treated and Sick NOD mice were collected, and 0.4×10^6^ purified CD4^+^ cells transferred into 4 week-old female NOD mice. Ninety percent of the CD4^+^CD25^+^ T cells from pancreatic LN (pLN) of CFA-treated NOD mice expressed Foxp3 (Figure 2D), suggesting that the great majority of the CD4^+^CD25^+^ cells that were transferred in the CD4^+^ population exhibited a regulatory cell phenotype. Blood glucose was monitored every week and diabetes incidence determined through 30 weeks of age. As shown in Figure 2E, CD4^+^ pancreatic LN cells from Non-Sick CFA-treated mice (NS) significantly delayed disease onset by 8 weeks (i.e., from 12 to 20 weeks) and significantly decreased the incidence of disease by comparison to untreated control mice (none). CD4^+^ pancreatic LN cells from Sick mice, on the other hand, actually accelerated disease onset and increased the incidence of disease from 85% in untreated control mice to 100%. To determine whether CD4^+^CD25^+^ regulatory T cells contributed to the transferred protection, CD4^+^ pancreatic LN cells from Non-Sick CFA-treated mice were depleted of CD25^+^ cells (NS CD4^+^CD25^−^) and transferred into 4-week-old NOD recipients. Unlike unfractionated CD4^+^ pancreatic LN cells from Non-Sick CFA-treated NOD mice, CD25^+^-depleted CD4^+^ cells from the same population were unable to protect against diabetes and, in fact, transfer of these cells increased the incidence of disease (to 100%) by comparison to untreated controls (Fig. 2E). These data suggest that CD4^+^CD25^+^ regulatory T cells play a significant role in disease prevention mediated by CFA, and CFA induces/activates CD4^+^CD25^+^ regulatory T cells that localize to the pancreatic LN and pancreas of NOD mice.

### The ability of NOD APC to activate CD4^+^CD25^+^ regulatory T cell function *in vitro* is enhanced by treatment with CFA

**Figure 3:**
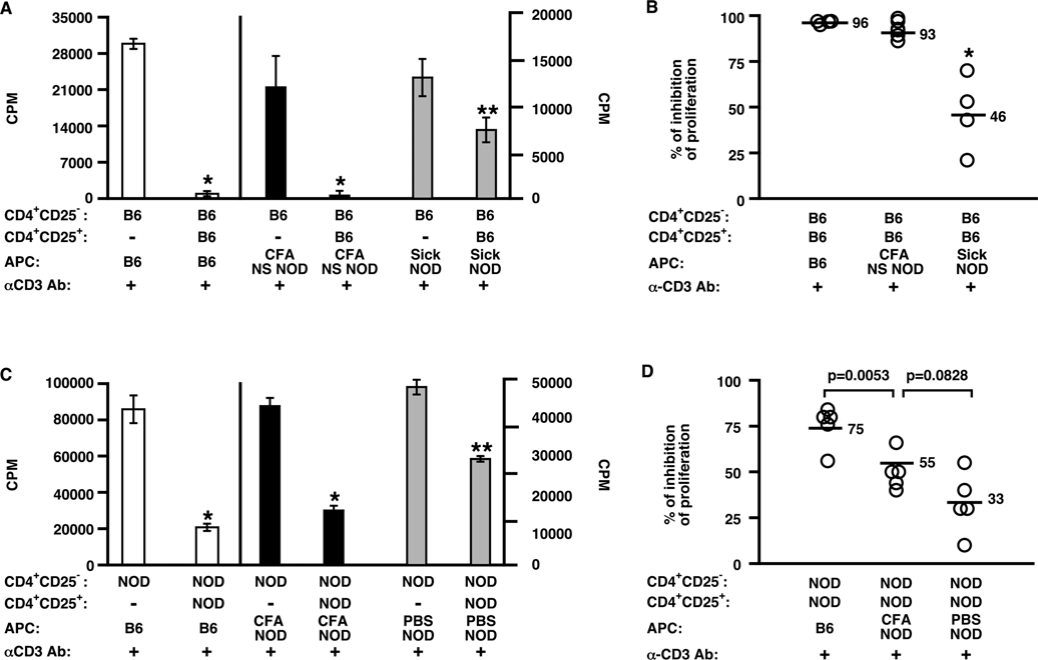
APC from CFA-treated NOD mice effectively induce regulatory cell function *in vitro*. CD4^+^CD25^−^ responder cells from B6 mice were cultured with irradiated spleen cells (APC) from individual 30 week-old B6, Non-Sick (NS) CFA-treated or Sick NOD mice, anti-CD3 and either with or without CD4^+^CD25^+^ cells from B6 mice (1∶1 regulatory∶responder ratio; A & B). CD4^+^CD25^−^ responder cells from NOD mice were cultured with irradiated spleen cells (APC) from individual 11 week-old NOD mice injected with PBS or CFA three weeks earlier or B6 mice, anti-CD3 and either with or without CD4^+^CD25^+^ cells from NOD mice (1∶1 regulatory∶responder ratio; C & D). Raw cpm data are shown for one representative animal (A & C), and percent inhibition is shown where each point represents an individual animal (B & D). * and ** indicate significant differences from the respective proliferation controls (i.e., responders alone; A & C), or B6 APC (B) at *p*<0.001 and p<0.05, respectively.

Since CFA appears to enhance CD4^+^CD25^+^ regulatory T cell function *in vivo* (Fig 2) and NOD APC are less effective stimulators of regulatory cells [16], at least *in vitro*, there is a possibility that CFA could be acting by activating NOD APC. Because a number of CFA-treated NOD mice will eventually develop diabetes and the mice that are going to develop diabetes will do so by 30 weeks of age, we analyzed Sick and Non-Sick NOD mice that were >30 weeks of age. We compared the ability of APC (irradiated spleen cells) from individual 30 week-old Non-Sick (NS) CFA-treated or Sick NOD mice, or B6 mice to activate B6 CD4^+^CD25^+^ regulatory cell function *in vitro*. To test NOD APC activity without the added complication of potentially defective NOD CD4^+^CD25^+^ cells and/or NOD CD4^+^CD25^−^ responder cells, we initially compared the ability of NOD APC from untreated Sick and CFA-treated Non-Sick NOD mice to induce B6 CD4^+^CD25^+^ regulatory cell activity *in vitro*. As shown in Figures 3A & B, CD4^+^CD25^+^ cells cultured in the presence of APC from Non-Sick CFA-treated mice (CFA NS NOD; Fig. 3A, black columns & 3B) suppressed proliferation of B6 CD4^+^CD25^−^ responder T cells in a manner similar to B6 APC (Fig. 3A, white columns & 3B), and to a significantly greater extent than APC from Sick NOD mice (Fig. 3A, gray columns & 3B). Interestingly, although APC from Non-Sick CFA-treated NOD mice activated regulatory cells more effectively than APC from untreated NOD mice, there were no differences in their ability to stimulate responder T cells, i.e., NOD APC are less effective at stimulating responders cells than B6 APC regardless of their treatment (Fig. 3A). Once we established that APC from Non-Sick CFA-treated NOD, but not untreated Sick NOD mice, could activate “normal” B6 CD4^+^CD25^+^ regulatory cells *in vitro*, we compared their ability to activate NOD CD4^+^CD25^+^ regulatory cells. As shown in Figures 3C & D, although not quite as effective as B6 APC, CFA-treated NOD APC (CFA NOD) were significantly better than PBS-treated NOD APC in inducing NOD CD4^+^CD25^+^ regulatory cell function *in vitro*, i.e., inhibition of proliferation was significantly increased in the presence of CFA-treated NOD APC versus PBS-treated NOD APC. Altogether, these data indicate that treatment with CFA enhances the ability of APC from NOD mice to activate CD4^+^CD25^+^ regulatory T cells.

### APC from CFA-treated NOD mice induce a fully activated CD4^+^CD25^+^ regulatory T cell phenotype

**Figure 4:**
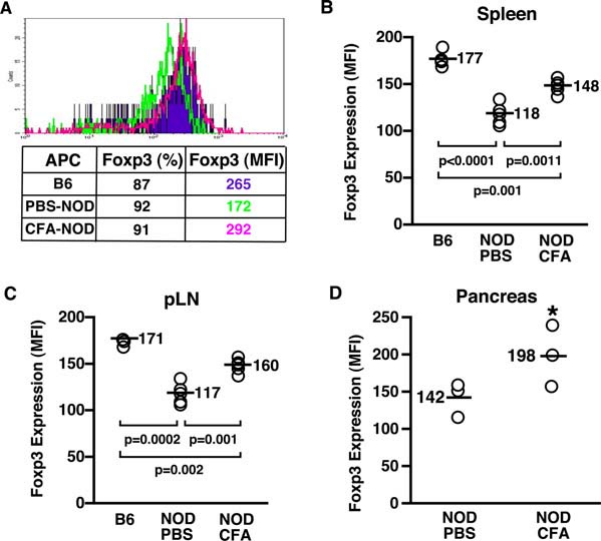
CFA treatment enhances Foxp3 expression by CD4^+^CD25^+^ regulatory T cells *in vivo*. CD4^+^CD25^+^ regulatory T cells were cultured for 24 hrs with APC (irradiated T cell-depleted spleen cells) pooled from 11 week-old B6 or NOD mice injected with PBS or CFA three weeks earlier and anti-CD3 antibody (A). Spleen (B), pancreatic LN (pLN) (C) and pancreas (D) were harvested from 11 week-old B6 or NOD mice injected with PBS or CFA three weeks earlier. Cells were labeled with anti-CD4, CD25 and Foxp3 antibodies and analyzed by FACS® gating on CD4^+^CD25^+^ cells (A–D). Each point represents an individual animal (B–D). An * indicates a significant difference from PBS-treated NOD (*p*<0.05).

Since APC from NOD mice treated with CFA can activate NOD CD4^+^CD25^+^ regulatory T cells effectively, we wondered whether CFA treatment also restored the ability of NOD APC to induce/maintain expression of markers important for regulatory cell function that we had found to be downregulated in NOD mice (Fig. 1). We first examined the expression of Foxp3 since we had found that NOD APC were defective at maintaining its expression in CD4^+^CD25^+^ cells *in vitro* (Table 2). CD4^+^CD25^+^ cells purified from the lymphoid organs of uninjected NOD mice were cultured overnight in the presence of T cell-depleted irradiated splenic cells from age-matched B6, Sick NOD or CFA-treated NOD mice and anti-CD3 antibody. Cells were harvested and Foxp3 expression by CD4^+^CD25^+^ cells evaluated. APC from CFA-treated NOD mice (pink line) increased Foxp3 expression to levels found with B6 APC (purple line), suggesting CFA enhanced the ability of NOD APC to maintain Foxp3 expression by CD4^+^CD25^+^ cells *in vitro* (Fig. 4A). We next examined whether CFA increased expression of Foxp3 *in vivo*. Spleen, pancreatic LN and pancreas were harvested from age-matched B6, PBS-treated NOD, and CFA-treated NOD mice 3 weeks after PBS or CFA injection, and Foxp3 expression by CD4^+^CD25^+^ T cells evaluated. CFA treatment of NOD mice produced a significant enhancement in Foxp3 expression by splenic and pancreatic LN (pLN) CD4^+^CD25^+^ cells compared to cells from untreated NOD mice (Fig. 4B & C). Similar results were found for cells harvested from the pancreas of CFA-treated NOD mice (Fig. 4D). These data suggest that CFA may mediate its protective effect by inducing or maintaining optimal levels of Foxp3 expression by CD4^+^CD25^+^ regulatory T cells in the periphery and at the inflammation sites via the activation of APC.

**Figure 5:**
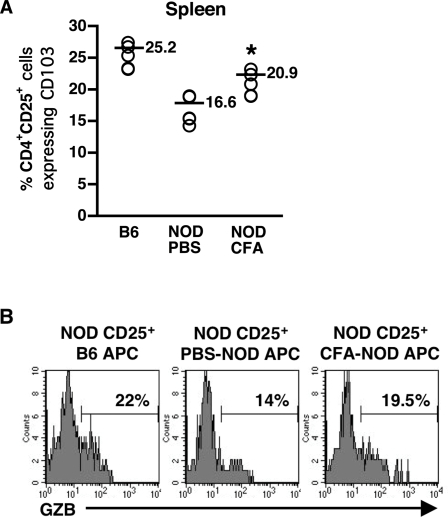
CFA treatment enhances the percentage of CD4^+^CD25^+^ regulatory T cells expressing CD103 and granzyme B. Spleen cells were harvested from 11 week-old B6 or NOD mice injected with PBS or CFA three weeks earlier. Cells were labeled with anti-CD4, CD25 and CD103 antibodies and analyzed by FACS® gating on CD4^+^CD25^+^ cells. Each point represents an individual animal. An * indicates a significant difference from PBS-treated NOD at *p*<0.05 (A). CD4^+^CD25^+^ T cells from 11 week-old NOD mice were pooled and cultured for 40 hrs with irradiated T cell-depleted spleen cells (APC) pooled (4–5 mice) from 11 week-old B6 or PBS- (NOD) or CFA-treated NOD (CFA-NOD) and anti-CD3 antibody. Cells were labeled with anti-CD4 and CD25 antibodies, and intracellularly labeled with anti-granzyme B antibody then analyzed by FACS® by gating on CD4^+^CD25^+^ cells. Representative results from one of three experiments are shown (B).

We also evaluated expression of other molecules associated with regulatory function that are decreased in either freshly harvested NOD CD4^+^CD25^+^ cells, i.e., CD103 [28], [29] and membrane-bound TGFβ [30], or after culture, i.e., granzyme B. While the percentages of CD4^+^CD25^+^ cells expressing CD103 were reduced in the spleen of NOD mice compared to those from B6 mice, they were significantly increased in the spleen of NOD mice treated with CFA compared to those from untreated NOD mice (Fig. 5A). Although not statistically significant, the percentages of CD4^+^CD25^+^ cells expressing mTGFβ tended to be higher (8.5+/−1.6% versus 5.9+/−1.3%) after CFA treatment. Finally, we examined the ability of APC from CFA-treated NOD mice to induce granzyme B (GZB) in CD4^+^CD25^+^ cells *in vitro.* GZB is a factor through which CD4^+^CD25^+^ regulatory T cells may mediate suppression *in vitro* [31]. CD4^+^CD25^+^ cells were pooled from multiple NOD mice, and cultured for 40 hrs in the presence of T cell-depleted irradiated splenic cells (APC) pooled from either B6, PBS-treated or CFA-treated NOD mice and anti-CD3. Cells were harvested and examined for intracellular GZB. The percentage of CD4^+^CD25^+^ cells expressing GZB was reduced when NOD APC (Fig. 5B, center panel) were used as stimulators by comparison to B6 APC (Fig. 5B, left panel). Interestingly, CFA increased the ability of NOD APC to induce GZB production by CD4^+^CD25^+^ cells (Fig. 5B, right panel). Altogether, the optimal expression of Foxp3, CD103 and granzyme B in CD4^+^CD25^+^ cells harvested from CFA-treated NOD mice or after culture with APC from CFA-treated NOD mice most likely reflects a fully activated regulatory cell phenotype, and further supports our hypothesis that CFA enhances the ability of NOD APC to activate CD4^+^CD25^+^ regulatory T cells.

## Discussion

NOD mice have major defects in immunoregulation [17], [32] and APC function [4]–[7] that could be involved in the development of diabetes. Our previous study suggested that defects in NOD APC could be directly responsible for the lower functional activity of CD4^+^CD25^+^ regulatory T cells in NOD mice [16]. In the current study, we show that activation of APC *in vivo* by treatment with CFA appears to significantly enhance the NOD APC's ability to activate CD4^+^CD25^+^ regulatory T cells, as indicated by increased regulatory activity both *in vivo* and *in vitro*. The enhanced regulatory activity associated with CFA treatment strongly correlated with restoration of an optimal regulatory cell phenotype, as reflected by increased Foxp3 expression and percentages of CD4^+^CD25^+^ T cells expressing CD103 and granzyme B, and most importantly, by the ability of this regulatory cell population to keep diabetogenic cells under control. Although CFA could have a direct effect on the regulatory cells themselves, our data suggest that its effect is at least in part mediated through enhancement of the ability of NOD APC to act on NOD CD4^+^CD25^+^ regulatory T cells at two related levels, i.e., maintaining them in a state of “readiness” by sustaining Foxp3 expression and producing optimal activation signals to stimulate regulatory activity.

The effect of CFA on diabetes prevention has been attributed to various cell types, including tolerogenic macrophages [24], suppressor T cells [25], and more recently NK cells [23]. Our study suggests, for the first time, that CD4^+^CD25^+^ regulatory T cells may play an important role in the control of diabetogenic cells and prevention of diabetes development in CFA-treated disease-free NOD mice. When CD4^+^ cells harvested from the pancreatic LN of disease-free CFA-treated NOD mice were depleted of CD25^+^ cells, the CD4^+^ cells lost their ability to prevent diabetes development after transfer into young NOD mice. Although, we have not ruled out the possibility that the CD25^+^ cells that appear to mediate protection from diabetes in our model could also include other types of regulatory cells, e.g., Th2 cells, the fact that 90% of the CD25^+^ cells in the pLN of CFA-treated NOD mice expressed Foxp3 tends to favor a traditional CD4^+^CD25^+^ regulatory rather than Th2 phenotype. More importantly, the enhanced ability of APC to activate CD4^+^CD25^+^ regulatory cells *in vitro* may reflect the mechanism by which CFA mediates regulatory cell activation *in vivo*. APC from NOD mice injected with CFA indeed exhibit an increased aptitude to stimulate CD4^+^CD25^+^ regulatory cells *in vitro*, as indicated by restored regulatory function, sustained Foxp3 expression and induction of granzyme B in CD4^+^CD25^+^ regulatory cells. Since there is increasing evidence of crosstalk between DC and NK cells [33], there is a possibility that CFA-activated APC may also activate NK cells that appear to be involved in CFA-mediated diabetes prevention [23].

It is not clear how CFA affects the ability of APC to activate regulatory cells. The enhancement of regulatory cell-inducing APC function in NOD mice treated with CFA is likely to be associated with an increase in expression/production of molecules important for the development and/or maintenance of CD4^+^CD25^+^ regulatory cells. There are many molecules associated with CD4^+^CD25^+^ regulatory cell development; CD28/B7 costimulation [34], [35] as well as IL-2 and TGFβ [36], [37], are crucial for the development, survival and maintenance of CD4^+^CD25^+^ regulatory cells as well as *in vitro* and *in vivo* conversion of CD4^+^CD25^−^ cells into CD4^+^CD25^+^ regulatory cells [37], [38]. Indeed, we have found that the percentage of NOD APC expressing B7-1 (including dendritic cells, macrophages and B cells), which is lower than that of B6 APC, is restored after CFA injection (data not shown). This is particularly significant, since B7-1 has been associated with tolerance in NOD mice, whereas B7-2 appears to be critical for induction of pathogenic responses [39].

Foxp3 is a molecule crucial for the development of CD4^+^CD25^+^ regulatory cells [11], [13]. Importantly, decreases in the levels of expression of Foxp3 appear to affect the ability of regulatory cells to control pathogenic cells. T cells with attenuated endogenous Foxp3 expression exhibit decreased suppressive activity and expression of regulatory cell signature genes, resulting in the development of an autoimmune syndrome similar to that of scurfy mice [15]. In addition, expression of Foxp3 mRNA at the single cell level has been shown to be impaired in CD4^+^CD25^+^ regulatory cells from NOD mice [40]. This decline in Foxp3 expression appears to start as early as 8 weeks of age and becomes more pronounced as the mice become sick, suggesting its association with the development of type 1 diabetes [40]. Our data confirm that the expression of Foxp3 at the protein level is decreased in NOD CD4^+^CD25^+^ cells, and that this decrease is not likely to be due to exposure to high glucose levels. Moreover, our data indicate that CFA-mediated protection strongly correlates with upregulation of Foxp3 expression by peripheral and pancreatic CD4^+^CD25^+^ regulatory cells, and increased infiltration of CD4^+^Foxp3^+^ cells into the pancreas. This is similar to a study showing that treatment with vasoactive intestinal peptide (VIP) upregulates Foxp3 in the pancreas, and restores tolerance by promoting the local differentiation and function of regulatory T cells [41]. We have also found that CFA administration enhances the percentages of CD4^+^CD25^+^ regulatory T cells expressing CD103, thought to be an activated effector/memory-like regulatory cell population [29] that exerts potent Ag-nonspecific suppression in the absence of TCR stimulation [42], and capable of sustained localization at inflammation sites [28], [29]. Culture of NOD regulatory cells with APC from CFA-treated NOD mice recapitulates the *in vivo* effect of CFA, as indicated by the increase in Foxp3 and granzyme B expression. The upregulation of Foxp3, CD103 and granzyme B expression may reflect restoration of a fully activated regulatory cell population, which may be crucial for the regulation of pathogenic cells and prevention of diabetes development. Upon stimulation with CFA, NOD APC may gain the ability to fully activate functional NOD regulatory cells that can successfully control pathogenic cells.

In conclusion, our study has provided “proof-of-concept” that manipulating the ability of APC to activate/induce CD4^+^CD25^+^ regulatory T cells is a potential strategy that can be used to prevent disease. However, finding the optimal and specific stimulus as well as identifying molecules that will target only regulatory cells, and not pathogenic cells, will be crucial for developing therapies to prevent and treat diabetes in humans. A cell wall molecule of *M. tuberculosis*, Mannose-capped lipoarabinomannan (ManLAM), has been shown to induce expansion of CD4^+^CD25^+^Foxp3^+^ regulatory T cells by binding to receptors expressed by APC [43]. ManLAM may, therefore, be a potential candidate for enhancing the ability of APC to induce preferentially regulatory cells.
